# Treatment with the Ferroptosis Inhibitor Ferrostatin-1 Attenuates Noise-Induced Hearing Loss by Suppressing Ferroptosis and Apoptosis

**DOI:** 10.1155/2022/3373828

**Published:** 2022-12-07

**Authors:** Peng-Wei Ma, Wei-Long Wang, Jia-Wei Chen, Hao Yuan, Pei-Heng Lu, Wei Gao, Xue-Rui Ding, Yu-Qiang Lun, Rui Liang, Zu-Hong He, Qian Yang, Lian-Jun Lu

**Affiliations:** ^1^Department of Otolaryngology Head and Neck Surgery, Tangdu Hospital, Air Force Medical University, Xi'an, China; ^2^Department of Otorhinolaryngology-Head and Neck Surgery, Zhongnan Hospital of Wuhan University, Wuhan, China; ^3^Department of Experimental Surgery, Tangdu Hospital, Air Force Medical University, Xi'an, China

## Abstract

Hair cell death induced by excessive reactive oxygen species (ROS) has been identified as the major pathogenesis of noise-induced hearing loss (NIHL). Recent studies have demonstrated that cisplatin- and neomycin-induced ototoxicity can be alleviated by ferroptosis inhibitors. However, whether ferroptosis inhibitors have a protective effect against NIHL remains unknown. We investigated the protective effect of the ferroptosis inhibitor ferrostatin-1 (Fer-1) on NIHL in vivo in CBA/J mice and investigated the protective effect of Fer-1 on tert-butyl hydroperoxide (TBHP)-induced hair cell damage in vitro in cochlear explants and HEI-OC1 cells. We observed ROS overload and lipid peroxidation, which led to outer hair cell (OHC) apoptosis and ferroptosis, in the mouse cochlea after noise exposure. The expression level of apoptosis-inducing factor mitochondria-associated 2 (AIFM2) was substantially increased following elevation of the expression of its upstream protein P53 after noise exposure. The ferroptosis inhibitor Fer-1was demonstrated to enter the inner ear after the systemic administration. Administration of Fer-1 significantly alleviated noise-induced auditory threshold elevation and reduced the loss of OHCs, inner hair cell (IHC) ribbon synapses, and auditory nerve fibers (ANFs) caused by noise. Mechanistically, Fer-1 significantly reduced noise- and TBHP-induced lipid peroxidation and iron accumulation in hair cells, alleviating ferroptosis in cochlear cells consequently. Furthermore, Fer-1 treatment decreased the levels of TfR1, P53, and AIFM2. These results suggest that Fer-1 exerted its protective effects by scavenging of ROS and inhibition of TfR1-mediated ferroptosis and P53-AIFM2 signaling pathway-mediated apoptosis. Our findings suggest that Fer-1 is a promising drug for treating NIHL because of its ability to inhibit noise-induced hair cell apoptosis and ferroptosis, opening new avenues for the treatment of NIHL.

## 1. Introduction

Hearing loss is the most common sensory disorder and seriously affects the quality of life of more than 1.5 billion people [1]. During social development, individuals are commonly exposed to industrial noise, traffic noise, and recreational noise of different intensities [2–5], which pose a serious threat to hearing. Noise-induced hearing loss (NIHL) is characterized by elevation of the hearing threshold caused by high-intensity or chronic noise and also leads to other physical and psychological disorders, including cardiovascular disease, sleep disturbance, and anxiety [3, 6–8].

High-intensity noise causes the death of cochlear hair cells, including inner hair cells (IHCs) and outer hair cells (OHCs) [9–11]. However, according to research on animal models and human autopsies, OHCs are more vulnerable to noise than IHCs [12–14]. Hair cells cannot regenerate in mammals, so loss of hair cells causes a permanent threshold shift (PTS) [15]. Although the detailed mechanism of noise-induced hair cell loss is still unclear because of its complexity, it is well accepted that metabolic damage, including oxidative stress damage, Ca^2+^ overload, and immune and inflammatory damage, are involved [9, 10, 16–18]. Upon exposure to loud noise, metabolism occurs at a very high level in the cochlea, resulting in the consumption of a large amount of energy and the production of reactive oxygen species (ROS) [12, 19, 20]. Oxidative stress damage to hair cells is believed to be the major mechanism of NIHL [21]. However, while some antioxidants have been verified to exert protective effects against NIHL in animal models [22, 23], there are almost no effective drugs for NIHL treatment.

Ferrostatin-1 (Fer-1), a synthetic antioxidant, is widely recognized for its ability to inhibit ferroptosis [24–26]. Ferroptosis, which was first proposed in 2012 by Dixon [27], has been identified as a new mode of cell death that is distinct from apoptosis and characterized by the deposition of iron and lipid peroxidation. Peroxidation of the cell membrane, which is composed of phospholipids, is the primary process underlying ferroptosis [28, 29]. Acyl-CoA synthetase long-chain family member 4 (ACSL4) is the key enzyme in the synthesis of polyunsaturated acyl tails (PL-PUFAs) and thus plays a crucial role in ferroptosis [30]. Transferrin receptor 1 (TfR1) transports iron into the cytoplasm and facilitates ferroptosis through the Fenton reaction. The glutathione peroxidase 4 (GPX4)-glutathione axis has been verified to eliminate peroxidized PL-PUFAs to inhibit ferroptosis [29]. Ferroptosis has been verified to be associated with the pathological processes of many diseases, including neurodegenerative disorders, stroke, traumatic brain injury, acute kidney injury, and cancer [25, 31, 32]. Recently, it was demonstrated that suppression of ferroptosis by Fer-1 or Liproxstatin-1 (another ferroptosis inhibitor) can effectively protect hair cells against cisplatin- or neomycin-induced ototoxicity [33–36]. However, whether Fer-1 treatment exerts cytoprotective effects against NIHL has not yet been reported.

Apoptosis-inducing factor mitochondria-associated 2 (AIFM2), which lacks a mitochondrial localization sequence but shares significant homology with apoptosis-inducing factor (AIF) and NADH oxidoreductases from bacteria to mammalian species, is the downstream target of P53 [37, 38]. AIFM2 can bind to DNA in a non-sequence-specific manner to induce caspase-independent apoptosis [39]. Some researchers have reported that AIFM2 plays an important role in acinar apoptosis during severe acute pancreatitis via the ATF6-P53-AIFM2 axis [40]. Recently, AIFM2 was reported to play a crucial role in suppressing ferroptosis in a ubiquinone-dependent manner [41, 42]. However, the mechanism by which AIFM2 regulates hair cell death is unclear.

In this study, we explored the protective effect of Fer-1 against NIHL using an in vivo mouse model and in vitro oxidative stress models (cochlear explants and HEI-OC1 cells). Furthermore, we explored the underlying mechanism of oxidative stress in these two models. We assessed the ability of systemic administration of Fer-1 to mitigate noise-induced auditory threshold elevation, OHC loss, cochlear synapse damage, and auditory nerve fiber (ANF) degradation. We hypothesized that Fer-1 protects hearing by scavenging ROS and inhibiting TfR1-mediated ferroptosis and P53-AIFM2 signaling pathway-mediated apoptosis.

## 2. Methods

### 2.1. Animals

CBA/J mice were purchased from The Jackson Laboratory and raised in our laboratory. All mice were given free access to water and food and were kept at 22 ± 1°C on a 12 : 12 h light-dark cycle. Female mice were chosen for this study. Baseline auditory brainstem responses (ABRs) were recorded at 9 weeks. Mice were exposed to noise when their weight reached 20~23 g at 10 weeks. All animal experimental procedures were approved by the Institutional Animal Care and Use Committee of Air Force Medical University.

### 2.2. Drug Administration

We dissolved Fer-1 (Sigma–Aldrich, SML0583) in DMSO (MP Biomedicals, 196055) to prepare a 190 mM stock solution and stored it at 4°C. The stock solution was diluted to the proper concentration (10 mg/kg) in saline containing 20% SBE-*β*-CD (MedChemExpress, HY-1703) immediately before injection. Mice in the Fer-1 treatment group received a total of 3 intraperitoneal (i.p.) injections and were used for immunohistochemistry, RNA extraction, and protein extraction. To determine ABR thresholds, we administered 2 extra i.p. injections to the mice the next day (in the a.m. and p.m.). Mice in the solvent-control group were injected with DMSO in 20% SBE-*β*-CD saline.

### 2.3. Auditory Brainstem Response (ABR) Measurement

We used ABR thresholds to evaluate mouse hearing function. ABRs were recorded 7 d before and 14 d after noise exposure as described previously [20]. Briefly, we properly anesthetized the mice by i.p. injection of 1% pentobarbital. Then, the mice were placed on a heating pad to maintain body temperature near 37°C. Three subdermal electrodes were inserted at the vertex of the skull (active), left mastoid region (reference), and right mastoid region (ground). ABRs were recorded at 8, 16, and 32 kHz with closed field tone pip stimuli by using a Tucker-Davis Technologies (TDT) RZ6 System (Alachua, FL, USA). Up to 1024 responses were averaged for each stimulus level. We used the ABR wave II to determine ABR thresholds for each frequency. The threshold was determined as the lowest intensity to evoke identifiable waves. All ABR measurements were conducted by the same experimenter.

### 2.4. Noise Exposure

Female CBA/J mice were exposed to noise individually by placing them in individual cages (5^∗^5^∗^15 cm^3^) installed in a sound-proof chamber. The mice were able to move without restraint during noise exposure. Broadband noise (BBN) of 2-20 kHz at 99~100 dB was generated by RZ6 Noise software. After amplification by a power amplifier (Crown, XLi800), the noise was output by 4 loudspeakers (CHUANGMU, CP-75A), which were positioned just above the cages. The level of noise around the sides of the cages was monitored by a sound level meter to ensure the uniformity of noise exposure. Control mice were exposed to the same conditions for 2 h without noise exposure.

### 2.5. Immunocytochemistry for Cochlear Surface Preparations

The detailed procedure was described in previous publications [20, 43]. Briefly, mice were deeply anesthetized and sacrificed 1 h after noise exposure. Then, the cochlea was removed and gently perfused with 4% paraformaldehyde (PFA). After fixation in 4% PFA overnight at 4°C, the cochleae were decalcified in 4% EDTA solution at 4°C for 5 d (the solution was changed every day). After decalcification, we carefully isolated the organ of Corti by removing surrounding tissues. Then, we cut the auditory epithelium into four turns (the apex, middle, base, and hook). Then, we adhered these specimens to a coverslip with Cell-Tak. The tissues were permeabilized with 1% Triton X-100 for 30 min at room temperature. After blocking with 10% normal donkey serum for 1 h at room temperature, the specimens were incubated with primary antibodies.

To visualize IHC synapses, the specimens were incubated with the following antibodies at 37°C overnight: Myosin VIIa antibody (Proteus Bioscience, sc-74516; 1 : 1000), CtBP2 IgG1 antibody (BD Biosciences, #612044; 1 : 500), and GluR2 IgG2a antibody (Millipore, #MAB397; 1 : 2000). After 3 washes with PBS, the specimens were incubated with Alexa Fluor 647-conjugated goat anti-mouse IgG1 (Invitrogen, A21240), Alexa Fluor 488-conjugated goat anti-mouse IgG2a (Invitrogen, A21131), and Alexa Fluor 568-conjugated goat anti-rabbit IgG (Invitrogen, A11036) diluted 1 : 500 for 1 h at 37°C in the dark and then at 4°C overnight.

To assess other targets, the specimens were incubated with the following antibodies at 4°C overnight: 4-HNE antibody (GeneTex, GTX17571; 1 : 100) and 3-NT antibody (Abcam, ab110282; 1 : 100). After 3 washes with PBS, the specimens were stained with Alexa Fluor 594-conjugated donkey anti-mouse IgG (Invitrogen, A21203) diluted 1 : 500 overnight at 4°C. After 3 washes with PBS, the specimens were stained with Alexa Fluor 488-conjugated phalloidin (Cytoskeleton, Inc. # PHDG1) diluted 1 : 60 and DAPI (Roche, 10236276001) diluted 1 : 1000 for 30 min at room temperature.

For hair cell counting, we incubated the specimens with Myosin VIIa antibody (Proteus Biosciences, #25-6790) diluted 1 : 1000 overnight at 4°C. After 3 washes with PBS, the specimens were stained with Alexa Fluor 594-conjugated donkey anti-rabbit IgG (Invitrogen, A21207) diluted 1 : 500 overnight at 4°C. After 3 washes with PBS, the specimens were stained with phalloidin and DAPI as described above in the dark for 30 min at room temperature.

Finally, all specimens were washed 3 times in in the dark with PBS and covered with another coverslip. Then, we sealed the edge with nail polish. Images were taken by a confocal laser scanning microscope (Olympus, FV1000).

### 2.6. Immunohistochemistry for Frozen Cochlear Sections

Frozen cochlear sections were obtained as previously described [19]. Briefly, mice were deeply anesthetized and sacrificed 1 h or 28 d after noise exposure. Then, the cochlea was harvested, fixed, and decalcified as described above. After gradient dehydration in 10%, 20%, and 30% sucrose, the specimens were embedded in optimal cutting temperature (OCT) compound. Then, 8 *μ*m-thick frozen sections were prepared by a Cryostat Microtome (Leica, CM1860). After appropriate permeabilization and blocking, the specimens were incubated with the following primary antibodies overnight at 4°C: Myosin VIIa antibody (Proteus Biosciences, #25-6790; 1 : 1000), Tuj1 antibody (GeneTex, GTX631836; 1 : 200), 4-HNE antibody (GeneTex, GTX17571; 1 : 100), 3-NT antibody (Abcam, ab110282; 1 : 100), Tuj1 antibody (Abcam, ab78078; 1 : 1000), P53 antibody (Abcam, ab26; 1 : 200), GPX4 antibody (Abcam, ab125066; 1 : 200), SOD2 antibody (GeneTex, GTX116093, 1 : 200), ACSL4 antibody (Abcam, ab155282; 1 : 100), and AIFM2 antibody (Santa Cruz Biotechnology, sc-377120; 1 : 50). After 3 washes with PBS, the specimens were incubated with Alexa Fluor 594-conjugated donkey anti-mouse IgG (Invitrogen, A21203) diluted 1 : 500 and Alexa Fluor 488-conjugated donkey anti-rabbit IgG (Invitrogen, A21206) diluted 1 : 500 overnight at 4°C. After 3 washes with PBS, the specimens were stained with DAPI diluted 1 : 1000 for 30 min at room temperature. Finally, the specimens were sealed appropriately. Images were taken by a confocal laser scanning microscope (Olympus, FV1000).

### 2.7. Whole Cochlear Tissue Homogenates and Western Blotting

Western blotting analysis was performed as previously described [19]. Briefly, mice were deeply anesthetized and sacrificed 1 h after noise exposure. Then, we harvested the cochlea on ice. We homogenized the cochlear tissues in working buffer (RIPA buffer containing protease inhibitor) by using a cryogenic grinder. The mixture was incubated on ice for 30 min and then centrifuged (12000 rpm, 20 min, 4°C). An aliquot of each supernatant was collected, and the protein concentration was determined by a BCA kit (Beyotime, PQ003); the remaining supernatants were added to loading buffer and stored at -20°C. Approximately 30 *μ*g of protein from each sample was separated by SDS–PAGE and then transferred onto a PVDF membrane (Millipore, IPVH00010). Then, the membrane was blocked with 5% skim milk in TBST for 1 h at room temperature. The membranes were incubated with the following primary antibodies: 4-HNE antibody (GeneTex, GTX17571; 1 : 2000), 3-NT antibody (Abcam, ab110282; 1 : 1500), P53 antibody (Abcam, ab26; 1 : 2000), TfR1 antibody (Invitrogen, 13-6890; 1 : 2000), GPX4 antibody (Abcam, ab125066; 1 : 2000), ACSL4 antibody (Abcam, ab155282; 1 : 4000), AIFM2 antibody (Santa Cruz Biotechnology, sc-377120; 1 : 500), SOD2 antibody (GeneTex, GTX116093, 1 : 2000), AIF antibody (GeneTex, GTX113306, 1 : 2000), BAX antibody (GeneTex, GTX109683, 1 : 2000), and GAPDH antibody (GeneTex, GTX100118; 1 : 5000) overnight at 4°C. Following 3 washes with TBST, the membranes were incubated with an appropriate secondary antibody diluted 1 : 2000 for 1 h at room temperature. After sufficient rinsing, the immunoreactive bands were visualized using Immobilon® Western Chemiluminescent HRP Substrate (Millipore, WBKLS0100).

### 2.8. RNA Extraction and qRT-PCR Analysis

The transcription levels of TfR1, GPX4, P53, AIFM2, ACSL4, AIF, SOD2, and BAX were measured by qRT-PCR. Mice (*n* = 3 for each group) were sacrificed 1 h after noise exposure (after the third injection) for the extraction of total RNA. The total RNA was extracted using the RNeasy Plus Mini Kit (QIAGEN, #74134) according to the manufacturer's instructions. The primer sequences for qRT-PCR analysis are listed in Table S1. The relative mRNA transcription levels were calculated by the 2^-*ΔΔ*CT^ method.

### 2.9. Cochlear Explant Culture and Drug Treatment

Cochlear explants were obtained as previously described [44]. In brief, Sprague–Dawley rat pups were sacrificed at P3, and the temporal bones were collected in Hank's balanced salt solution. Then, we carefully isolated the auditory epithelium. The specimens were placed on the surface of a premade collagen gel immersed in 1 mL serum-free BME medium (SFM). Then, the cochlear explants were incubated in an incubator (37°C, 5% CO_2_) for 4 h, followed by the addition of 1 mL SFM. The next day, the cochlear explants in the Fer-1 control group and Fer − 1 + tert − butyl hydroperoxide (TBHP) group were pretreated with 40 *μ*M Fer-1 for 24 h. On the third day, cochlear explants in the TBHP group and Fer − 1 + TBHP group were treated with 100 *μ*M TBHP (Sigma–Aldrich, 458139) for 3 h. Then, the cochlear explants in all groups were fixed, permeabilized, and blocked as mentioned above. The cochlear explants were incubated with Myosin VIIa to label hair cells. Finally, all specimens were stained with DAPI and sealed.

### 2.10. Cell Culture and Cell Viability Analysis

HEI-OC1 cells were cultured in DMEM (HyClone, SH30243) containing 10% FBS (Gibco, 10099) and 1% penicillin and streptomycin solution (HyClone, SH40003) at 37°C in 5% CO_2_. For cell viability analysis, we used a cell counting kit-8 (CCK-8) according to the manufacturer's instructions (Plant Chem Medicine, PC-1050-1). Briefly, cells were seeded in 96-well plates (5000 cells/well) and incubated for 24 h. Then, the cells were pretreated with Fer-1 for another 24 h. The cells were treated with 250 *μ*M TBHP for 90 min, and then the culture medium was replaced with 10 *μ*L of CCK-8 reagent in 90 *μ*L of fresh DMEM. The cells were incubated for 2 h at 37°C, and the absorbance was determined at 450 nm by a plate reader (BioTek, EPOCH™).

### 2.11. Intracellular ROS and Lipid Peroxidation Assessment

The MDA level was measured to evaluate lipid peroxidation (Dojindo, M496). Briefly, mice were deeply anesthetized and perfused with 0.9% saline 1 h after noise exposure. Then, the cochlea was removed rapidly on ice, and cochlear tissues were homogenized in PBS containing antioxidants by a cryogenic grinder. After centrifugation (10000 × g, 5 min, 4°C), 200 *μ*L aliquots of the supernatants were transferred to a new tube. Then, we added 200 *μ*l of lysis buffer to the new tube. Following vortexing, we incubated the reaction mixture at room temperature for 5 min. After that, 300 *μ*L of working solution was added to the tube, and the samples were incubated at 95°C for 15 min. After being cooled in an ice bath for 5 min, all tubes were centrifuged at 10000 × g for 10 min at 4°C. Finally, we added 200 *μ*L of the reaction mixture to a 96-well plate and measured the absorbance at 532 nm by a microplate reader (BioTek, EPOCH™).

We used Liperfluo (DojinDo Laboratories, L248) to evaluate lipid peroxidation in cochlear explants treated with TBHP. Briefly, the samples were washed twice with SFM. Then, 40 *μ*M Liperfluo working solution was added to the samples, and the samples were incubated for 30 min in a 37°C incubator. After being washed with SFM twice, the samples were fixed for immunofluorescence staining as described above.

We used DCFH-DA (Sigma–Aldrich, D6883) to evaluate ROS accumulation in HEI-OC1 cells. Briefly, HEI-OC1 cells were washed with cold PBS twice. Then, 50 *μ*M DCFH-DA working solution was added to the samples, and the samples were incubated for 30 min in a 37°C incubator. Finally, the samples were fixed in 4% PFA and labeled with DAPI.

### 2.12. Intracellular Iron Assessment

We used FerroOrange (Dojindo Laboratories, F374), a novel fluorescent probe that enables the labeling of intracellular Fe^2+^, to evaluate changes in Fe^2+^ concentrations in cochlear explants treated with TBHP. Briefly, cochlear explants were washed with SFM twice. Then, 5 *μ*M FerroOrange working solution was added to the samples, and the samples were incubated for 30 min in a 37°C incubator. Finally, the samples were fixed for immunofluorescence staining as described above. For cell samples, the concentration of FerroOrange working solution was 2 *μ*M.

### 2.13. Flow Cytometry

Apoptosis analysis was carried out with a flow cytometry assay using an Annexin V-FITC/PI kit (BD Biosciences Pharmingen, 556547). Briefly, HEI-OC1 cells were washed with cold PBS and resuspended in 1 × binding buffer at a concentration of 1 × 10^6^ cells/mL. Then, Annexin V-FITC and propidium iodide were added and incubated for 15 min at room temperature in the dark. Finally, the samples were analyzed with a Beckman flow cytometer within 1 h.

### 2.14. TUNEL Assay

We used a TUNEL assay kit (Roche, 12156792910) to evaluate DNA strand breaks in cells undergoing apoptosis. Briefly, HEI-OC1 cells were washed with cold PBS and fixed in 4% PFA. After permeabilization, 100 *μ*L of TUNEL reaction mixture was added to each sample for 60 min of incubation at 37°C. Finally, the samples were labeled with DAPI.

### 2.15. Semiquantitative Analysis of Fluorescence Signals

ImageJ software (version 1.53a, USA) was used for semiquantification of fluorescence signals. Briefly, the fluorescent intensity of 4-HNE and 3-NT was measured from original confocal images from each experiment taken under identical settings. Phalloidin (green) and DAPI (blue) counterstaining was performed to identify OHCs.

The fluorescence intensity inside OHCs was measured in the base turn (~32 kHz) of the cochlea, and the average fluorescence intensity per cell was then calculated. Finally, the relative values were calculated by normalization to the value of the control group.

### 2.16. Hair Cell and Synapse Counts

After the last ABR test, we harvested the cochlea and prepared cochlear surface preparations. Images were captured by a 10 × objective with an Olympus confocal microscope. Hair cells were counted from the apex through the base to calculate the loss rate as described by Müller's [45]. Then, we generated a cytocochleogram to determine the percentage of hair cell loss [11].

For the quantification of IHC ribbon synapses, images captured with a 60 × objective (zoom × 2) on an Olympus confocal microscope were analyzed in the 120 *μ*m segment (containing approximately 12 IHCs) of the surface preparation corresponding to frequencies of approximately 16 kHz. Only regions in which CtBP2 (red) and GluR2 (green) were colocalized and were considered paired (functional) synapses. The total number of paired synapses was counted, and the average number of paired synapses per IHC was calculated.

### 2.17. Coimmunoprecipitation (coIP) Assay

For detecting the interaction between proteins, HEK293 cells were transfected with FLAG-P53 and cultured at 37°C in 5% CO_2_ overnight. The next day, HEK293 cells were lysed with IP buffer (containing protease inhibitor) for 20 min on ice. After centrifugation (12000 rpm, 20 min, 4°C), the supernatant was collected. The magnetic beads (Millipore, LSKMAGG02) were washed with PBS containing 0.1% Tween 20 (PBST) and then incubated with anti-FLAG M2 (Sigma–Aldrich, SLBT7654) at room temperature for 10 min (continuous mixing). After 3 times washes with PBST, the beads were incubated with protein supernatant at 4°C with continuous mixing overnight. The next day, the beads were washed 3 times with PBST and the protein complex was eluted from the beads with elution buffer. Finally, the sample was analyzed by immunoblotting.

### 2.18. Perilymph Extraction and Sample Preparation

Perilymph extraction and sample preparation was performed as previously described [19]. Mice were injected intraperitoneally with 10 mg/kg Fer-1. 15 min, 1 h, and 3 h after the injection, these mice were decapitated and the cochleae were removed. Then perilymph (approximately 1 *μ*L) was collected using a custom tube by piercing the round window membrane of the cochlea. After that, the collected perilymph was added into 500 *μ*L of methanol. After vortexing, the sample was filtered with a 0.22 *u*m filter. The liquid was harvested and used for liquid chromatography tandem mass spectrometry (LC-MS/MS) analysis.

### 2.19. Chromatographic Separation and MS/MS Detection

The LC-MS/MS analysis was performed with the mass spectrometer (SHIMADZU, LCMS-8050, Japan) as previously described [19]. The Agilent C18 column (50 × 2.1 mm i.d., 1.9 *μ*m particle size) was used to perform chromatographic separation. The sample injection volume was 5 *μ*L. The column temperature was maintained at 35°C. Then, a gradient of mobile phase A (0.1%(v/v) formic acid in 5 mM ammonium acetate) and mobile phase B (acetonitrile) was run at 0.4 mL/min. The MS/MS analysis was performed in the positive electrospray ionization (ESI+) mode. The source temperature was kept at 400°C. The ion spray voltage was 4500 V. The detection of the ions was operated in the multiple reaction monitoring (MRM) mode, monitoring transitions of *m*/*z* 263.35 → 180.95 for Fer-1. The quantification of the Fer-1 in the perilymph was analyzed by calculating the peak area ratios using the external standard.

### 2.20. Statistics

Statistical analyses were conducted using GraphPad Prism software (version 8.0, USA). Comparisons among groups were analyzed by one-way analysis of variance (ANOVA), and Sidak's multiple comparisons test was used to assess the difference between two groups. OHC loss along the length of the cochlear spiral was analyzed with two-way ANOVA followed by Sidak's multiple comparisons test. A *p* value of <0.05 was considered statistically significant. All the data are presented as the mean ± standard deviation (SD).

## 3. Results

### 3.1. Fer-1 Entered Inner Ear after Systemic Administration

In order to investigate whether Fer-1 existed in the inner ear after the systematic administration, we analyzed the concentration of Fer-1 in the perilymph of mouse cochlea using liquid chromatography tandem mass spectrometry (LC-MS/MS). The perilymph was extracted 15 min, 1 h, and 3 h after the injection and used for the detection and quantification of Fer-1. As shown in Figure S3, the retention time of the Fer-1 in the perilymph was 5.761 min. The mass-to-charge ratio (*m*/*z*) of the parent ion was 263.35, which was consistent with the relative molecular mass of Fer-1 getting a hydrogen ion. At 15 min, 1 h, and 3 h after injection, the concentrations of the Fer-1 in perilymph of mouse cochlea were 2.077, 0.301, and 0.122 *μ*g/L, respectively. These results indicated that Fer-1 could enter the inner ear.

### 3.2. Administration of Fer-1 Attenuated Noise-Induced ABR Threshold Shifts

**Figure 1:**
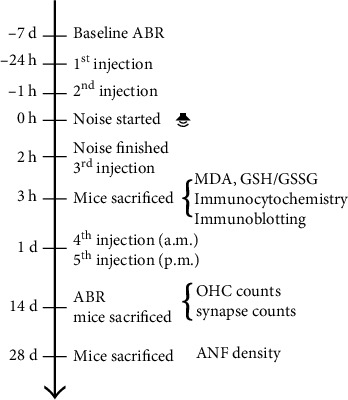
Experimental procedure. The baseline ABR of mice was measured 7 d before noise exposure. Mice were administered Fer-1 or vehicle (saline and 20% SBE-*β*-CD) by i.p. injection 24 h and 1 h before noise exposure. Then, mice were exposed to 99~100 dB SPL 2-20 kHz BBN for 2 h. The mice received the third injection immediately after the noise was turned off. Mice used for the analysis of oxidative stress levels were sacrificed 1 h after the end of noise exposure. Mice used for analysis of the effects of Fer-1 treatment on auditory threshold shifts were administered two more i.p. injections the next day (in the a.m. and p.m.). After ABR testing 14 d following noise exposure, the mice were sacrificed to evaluate OHC loss and IHC synapse loss. Twenty-eight days after noise exposure, the mice were sacrificed for evaluation of ANF degeneration. BBN: broadband noise.

**Figure 2:**
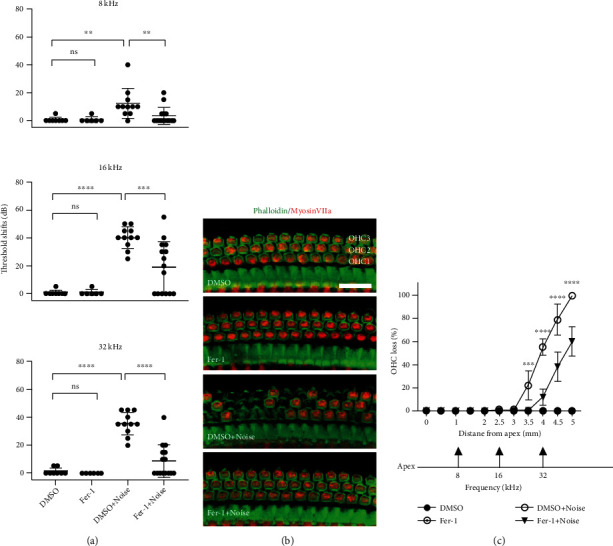
Fer-1 treatment alleviates noise-induced auditory threshold elevation and OHC loss. (a) Fer-1 treatment considerably reduces noise-induced mouse ABR threshold shifts. The data are shown as individual points and the means ± SD of each group. ^∗∗∗∗^*p* < 0.0001; ^∗∗∗^*p* < 0.001; ^∗∗^*p* < 0.01; ns: not significant. (b) Representative images show noise-induced OHC loss. Pictures were taken from the basal turn of the cochlea (~4.0 mm from the apex). Scale bar = 20 *μ*m. (c) Cochlear OHC loss assessed 14 d after noise exposure presented as a percentage. Fer-1 treatment markedly alleviated noise-induced OHC loss. ^∗∗∗∗^p < 0.0001; ^∗∗∗^p < 0.001.

To evaluate the protective effect of Fer-1, the experimental procedure shown in Figure 1 was performed. The hearing function of the four groups of mice (DMSO control, Fer-1 control, DMSO + noise, and Fer − 1 + noise) was tested by measuring the ABR at 8, 16, and 32 kHz 7 d before (baseline ABR) and 14 d after noise exposure. The ABR was significantly different among four groups of mice at 8 kHz (*F* (3, 35) = 5.902, *p* = 0.0023), 16 kHz (*F* (3, 35) = 19.99, *p* < 0.0001), and 32 kHz (*F* (3, 35) = 34.39, *p* < 0.0001), as analyzed by one-way ANOVA. There was no significant difference in the baseline ABR threshold among the groups (Figure S1). At 8 kHz (*p* = 0.0051), 16 kHz (*p* < 0.0001), and 32 kHz (*p* < 0.0001), the ABR threshold was significantly increased in the noise exposure group compared with the control group (Figure 2(a)). In comparison with the group of mice exposed to noise (DMSO + noise group vs. Fer − 1 + noise group), the Fer-1-treated group exhibited significant attenuated of noise-induced auditory threshold shifts at 8 kHz (*p* = 0.0134), 16 kHz (*p* = 0.0008), and 32 kHz (*p* < 0.0001) (Figure 2(a)). Moreover, the ABR threshold of mice treated with Fer-1 did not differ from that of mice in the DMSO group. These results indicated that administration of Fer-1 was able to attenuate noise-induced ABR threshold shifts.

### 3.3. Administration of Fer-1 Prevented OHC Loss Induced by Noise Exposure

To further evaluate the protective effect of Fer-1 against noise exposure, we then counted the number of hair cells, which were stained with phalloidin (green) and labeled with Myosin VIIa (red), on cochlear surface preparations obtained 14 d after noise exposure. Loss of the cell body (Myosin VIIa) and cytoskeleton (phalloidin) was considered to indicate OHC loss (Figure 2(b)). Consistent with the findings reported in other literatures [11], our results showed that the loss of OHCs started 3.5 mm from the apex and reached nearly 100% at the end of the cochlear epithelium. Five i.p. injection of 10 mg/kg significantly reduced OHC loss caused by noise, as analyzed by two-way ANOVA followed by posthoc tests (for detailed values, see Table 1 for Figure 2(c)). These results indicated that Fer-1 was able to prevent OHC death after noise exposure.

### 3.4. Treatment with Fer-1 Mitigated Noise-Induced Oxidative Stress in the Cochlea following Noise Exposure

**Figure 3:**
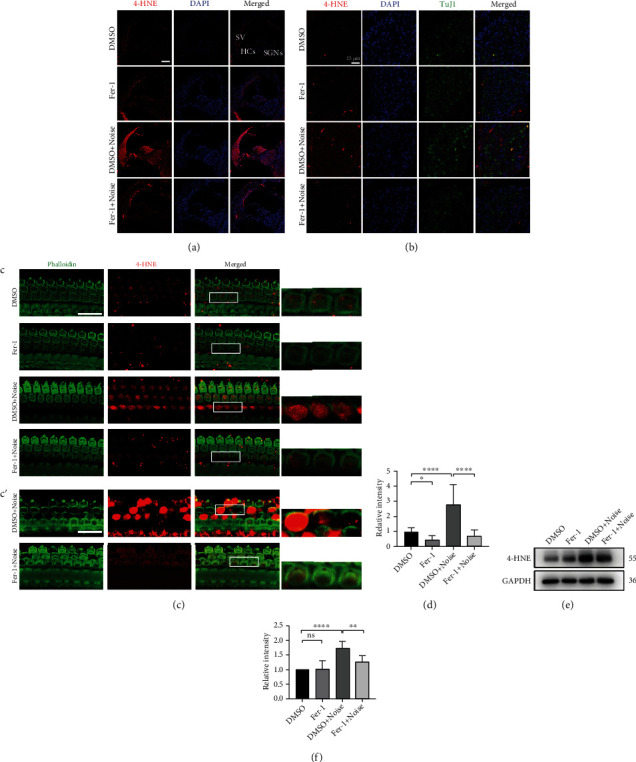
Fer-1 treatment reduces the level of the lipid peroxidation product 4-HNE following noise exposure. (a) Representative pictures of noise-induced elevation of 4-HNE levels (red) in frozen cochlear sections. Scale bar = 100 *μ*m. HCs: hair cells; SV: stria vascularis; SGNs: spiral ganglion neurons. (b) Representative pictures of 4-HNE (red) in SGNs (labeled with TuJ1, green) in frozen cochlear sections. Scale bar = 25 *μ*m. (c) Representative pictures of 4-HNE (red) in OHCs (stained with phalloidin, green) in surface preparations. Scale bar = 20 *μ*m. (c′) Representative pictures of 4-HNE in OHCs at the more basal region. The pictures above were taken from the base turn of the cochlea 1 h after noise exposure. (d) Quantification of relative 4-HNE immunolabeling intensity in grayscale in OHCs normalized to DMSO control mice (Figure 3(c)); *n* = 3 for each condition. (e) Representative blots show that the expression level of 4-HNE was increased in the noise exposure group compared with the vehicle control groups 1 h after noise exposure. Fer-1 treatment markedly alleviated the increase in 4-HNE level caused by noise. (f) Semiquantification of the band density (Figure 3(e)); *n* = 3 for each condition. The data are presented as the means ± SDs. ^∗∗∗∗^*p* < 0.0001; ^∗∗^*p* < 0.01; ^∗^*p* < 0.05; ns: not significant.

**Figure 4:**
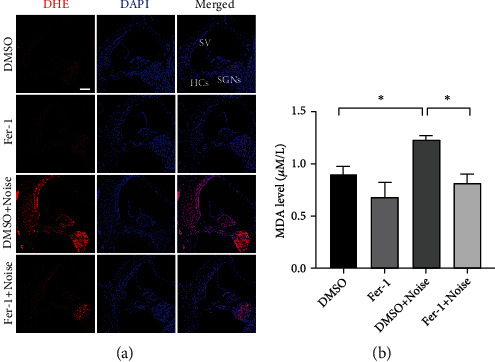
Treatment with Fer-1 mitigates the elevation of ROS and MDA levels in the cochlea. (a) Representative pictures of noise-induced elevation of DHE levels (red) in frozen cochlear sections. Scale bar = 100 *μ*m. SV: stria vascularis; SGNs: spiral ganglion neurons; HCs: hair cells. (b) The cochlear MDA level 1 h after noise; *n* = 3 for each condition. ^∗^*p* < 0.05.

Excessive accumulation of ROS is a primary cause of NIHL and hearing loss because OHCs are one of the most vulnerable structures to damage, as reported by own laboratory and others [19, 20]. Excessive ROS oxidize lipids to induce lipid peroxidation, which can be evaluated by measuring 4-HNE and MDA levels. To assessed whether the administration of Fer-1 prevented NIHL via inhibition of lipid peroxidation, we performed immunolabeling for 4-HNE. As shown in Figure 3(a), after noise exposure, the intensity of 4-HNE was substantially increased in the cochlea, including hair cells, stria vascularis (SV), and spiral ganglion neurons (SGNs). Compared with DMSO, Fer-1 treatment significantly alleviated lipid peroxidation caused by noise-induced excessive ROS production. To further assess oxidative stress in SGNs, we observed SGNs at higher magnification (Figure 3(b)). 4-HNE fluorescence was evenly distributed in the cytoplasm. As shown in Figure 3(b), Fer-1 treatment decreased the intensity of 4-HNE fluorescence. Since loss of OHCs is the major cause of hearing loss, we evaluated oxidative stress in OHCs in cochlear surface preparations 1 h after noise exposure. As shown in Figure 3(c), after noise exposure, the 4-HNE fluorescence intensity was increased approximately 2.5-fold in OHCs, which were labeled with phalloidin-488. Administration of Fer-1 significantly reduced the 4-HNE intensity in OHCs in both the presence (DMSO + noise vs. Fer − 1 + noise, *p* < 0.0001) and absence (DMSO vs. Fer-1, *p* = 0.0366) of noise exposure (Figures 3(c) and 3(d)). It is worth noting that we did not observe OHC loss in this region 1 h after noise exposure, but OHC loss accompanied by elevation of 4-HNE levels was observed in the more basal region (Figure 3(c)'). Administration of Fer-1 significantly reduced the loss of OHCs and 4-HNE intensity in this more basal region. We then assessed 4-HNE protein expression levels in the cochlea after noise exposure in the presence or absence of Fer-1. As shown in Figure 3(e) and Figure 3(f), the expression level of 4-HNE was significantly increased (DMSO vs. DMSO + noise, *p* < 0.0001) following noise exposure. Treatment with Fer-1 significantly reduced (DMSO + noise vs. Fer − 1 + noise, *p* = 0.0036) the expression level of 4-HNE in the cochlea after noise exposure, but the expression level was still higher than that in the control group. Finally, we measured MDA levels in the four groups 1 h after noise exposure, and the change was similar to that in 4-HNE levels (DMSO vs. DMSO + noise, *p* = 0.0335; DMSO + noise vs. Fer − 1 + noise, *p* = 0.0102) (Figure 4(b)).

**Figure 5:**
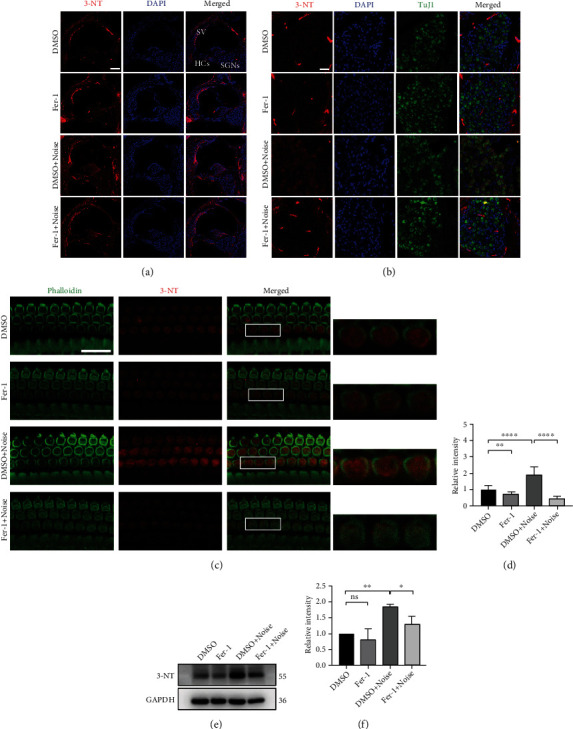
Fer-1 treatment reduces the levels of the protein nitration product 3-NT following noise exposure. (a) Representative pictures of noise-induced elevation of 3-NT levels (red) in frozen cochlear sections. Scale bar = 100 *μ*m. HCs: hair cells; SV: stria vascularis; SGNs: spiral ganglion neurons. (b) Representative pictures of 3-NT (red) in SGNs (labeled with TuJ1, green) in frozen cochlear sections. Scale bar = 25 *μ*m. (c) Representative pictures of 3-NT (red) in OHCs (stained with phalloidin, green) in surface preparations. Scale bar = 20 *μ*m. The pictures above were taken from the base turn of the cochlea 1 h after noise exposure. (d) Quantification of relative 3-NT immunolabeling intensity in grayscale in OHCs normalized to DMSO control mice (Figure 5(c)); *n* = 3 for each condition. (e) Representative blots show that the expression level of 3-NT was increased in the noise exposure group compared with the vehicle control group 1 h after noise exposure. Fer-1 treatment markedly alleviated the increase in the level of 3-NT caused by noise. (f) Semiquantification of the band density (Figure 5(e)); *n* = 3 for each condition. The data are presented as the means ± SDs. ^∗∗∗∗^*p* < 0.0001; ^∗∗^*p* < 0.01; ^∗^*p* < 0.05; ns: not significant.

The accumulation of ROS also causes protein nitration, which can be detected by 3-NT. Therefore, we evaluated the level of 3-NT in the cochlea under different conditions. The results were consistent with the changes in 4-HNE levels. Specifically, noise exposure caused a marked increase in 3-NT levels in the cochlea. The increase in the level of 3-NT was inhibited by administration of Fer-1 (Figure 5(a)–5(f)).

Dihydroethidium (DHE) is a fluorescent dye that can detect superoxide ions. We also evaluated noise-induced cochlear oxidative stress by directly detecting DHE. As shown in Figure 4(a), treatment with Fer-1 markedly reduced the intensity of DHE fluorescence induced by noise exposure. Taken together, these data indicated that Fer-1 alleviated noise-induced cochlear oxidative stress by scavenging ROS and thereby mitigating lipid peroxidation and protein nitration.

### 3.5. Fer-1 Treatment Reduced Noise-Induced Loss of IHC Ribbon Synapses and Degeneration of ANFs

**Figure 6:**
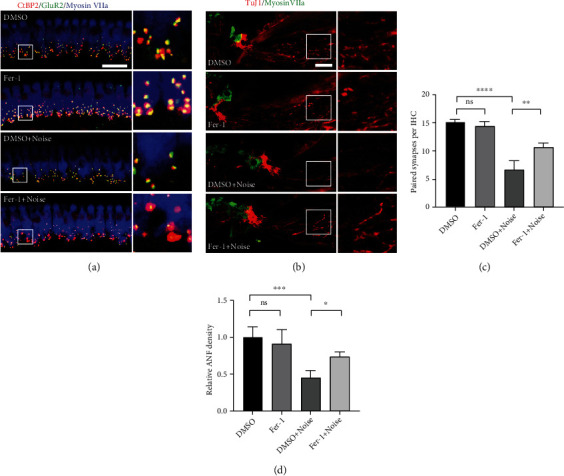
Fer-1 administration alleviates IHC synapse loss and ANF degeneration after noise exposure. (a) Representative pictures of ribbon synapse loss in IHCs caused by noise (presynaptic structures, red; postsynaptic structures, green; IHCs, blue). Pictures were taken of the middle turn of the cochlea, which corresponded to ~16 kHz, scale bar = 20 *μ*m. (b) Representative pictures of ANF degeneration caused by noise (ANFs, red; HCs, green). Scale bar = 20 *μ*m. (c) Quantification of “paired” synapses in IHCs corresponding to ~16 kHz 14 d after noise exposure; *n* = 3 for each condition. (d) Quantification of TuJ1-immunolabeled ANFs 28 d after noise exposure; *n* = 3 for each condition. ^∗∗∗∗^*p* < 0.0001; ^∗∗∗^*p* < 0.001; ^∗∗^*p* < 0.01; ns: not significant.

Recent studies have shown that ribbon synapses located between IHCs and SGNs are the primary structures affected by noise [46, 47]. To explore the protective effect of Fer-1 against IHC ribbon synapse loss caused by noise, the number of paired ribbon synapses was quantified 14 d after noise exposure as previously reported [19]. Presynaptic structures were labeled with CtBP2 (red), and postsynaptic structures were labeled with GluR2 (green). Regions in which CtBP2 and GluR2 (yellow) were colocalized were identified as functional (paired) synapses. The region of cochlear surface preparations in which IHC synapses were counted corresponded with approximately 16 kHz. As shown in Figure 6(a), the number of paired synapses was significantly reduced 14 d after noise exposure (*p* < 0.0001). Treatment with Fer-1 markedly reduced the loss of paired synapses induced by noise exposure, and the difference was statistically significant (*p* = 0.0040) (Figure 6(c)). It is worth noting that CtBP2-positive presynaptic structures seemed to be larger following Fer-1 treated, especially after noise exposure. These larger presynaptic structures indicated incomplete repair of synapses.

In addition, when ANFs are disconnected from IHCs, degeneration inevitably occurs [46]. Therefore, we evaluated the protective effects of Fer-1 treatment against ANF degeneration by measuring the fiber density 28 d after noise exposure. As shown in Figures.  6(b) and 6(d), the density of ANFs was significantly reduced 28 d after noise exposure (*p* = 0.0004). Treatment with Fer-1 significantly reduced ANF degeneration caused by noise (*p* < 0.0001).

### 3.6. Treatment with Fer-1 Alleviated TBHP-Induced Cochlear Explant Hair Cell and HEI-OC1 Cell Damage

**Figure 7:**
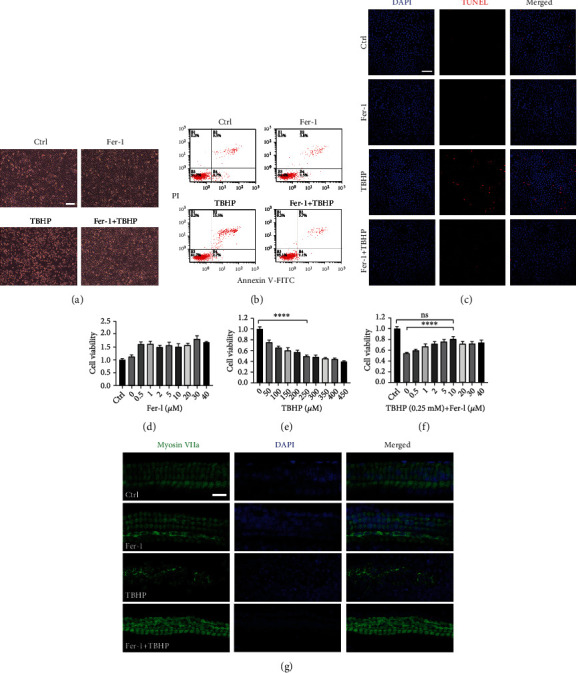
Treatment with Fer-1 alleviates TBHP-induced cochlear explant and HEI-OC1 cell damage. (a) Representative images of HEI-OC1 cells treated with TBHP and Fer-1. Scale bar = 100 *μ*m. (b) Flow cytometry analysis of cell apoptosis of the cells in Figure 7(a). (c) DAPI and TUNEL double labeling of apoptotic cells after treatment with TBHP and Fer-1. Scale bar = 100 *μ*m. (d–f) Cell viability was assessed after treatment with different concentrations of Fer-1 and TBHP. (g) Pretreatment with Fer-1 alleviates TBHP-induced cochlear hair cell death in the middle turn. Scale bar = 20 *μ*m. ^∗∗∗∗^*p* < 0.0001; ns: not significant.

To further verify the protective effect of Fer-1, in vitro oxidative stress experiments were performed in cochlear explants and HEI-OC1 cells. And oxidative stress damage was induced by TBHP for the *in vitro* experiments. HEI-OC1 cell line is an immortalized cochlear sensory epithelial cell line that expresses multiple hair cell markers, so it is widely used for studying hair cell pathology. We first tested the effects of different concentrations (0 ~ 40 *μ*M) of Fer-1 on the cells and found that Fer-1 had no cytotoxicity (Figure 7(d)). We then tested the viability of HEI-OC1 cells exposed to a series of doses of TBHP (0 ~ 450 *μ*M) (Figure 7(e)). CCK-8 assay indicated that treatment with TBHP at a concentration of 250 *μ*M for 90 min reduced the cell viability to about 50%. And we further assessed the impact of a series of doses of Fer-1 against the effects of 250 *μ*M TBHP. We found that treatment with Fer-1 prevented cell death after TBHP treatment in a dose-dependent manner, with the most effective concentration being 10 *μ*M (Figure 7(f)). As shown in Figure 7(a), pretreatment with 10 *μ*M Fer-1 for 24 h prior to 250 *μ*M TBHP exposure for 120 min markedly reduced cell death. To further verify that Fer-1 treatment reduced TBHP-induced HEI-OC1 cell death, we performed flow cytometry, and the results showed substantially fewer apoptotic cells in Fer − 1 + TBHP group than in TBHP group (Figure 7(b)). In addition, after exposure to 250 *μ*M TBHP for 120 min, a large number of TUNEL-positive cells were observed. However, there were no TUNEL-positive cells in Fer − 1 + TBHP group (Figure 7(c)). Moreover, we tested the protective effect of Fer-1 against oxidative stress damage at the organ level. As shown in Figure 7(g), treatment of cochlear explants with 100 *μ*M TBHP for 3 h caused severe damage to hair cells. Pretreatment with 40 *μ*M Fer-1 for 24 h markedly alleviated hair cell loss. These data indicated that Fer-1 treatment significantly alleviated TBHP-induced cochlear explant hair cell and HEI-OC1 cell death.

### 3.7. Treatment with Fer-1 Alleviated TBHP-Induced Oxidative Stress and Iron Accumulation in Cochlear Explants and HEI-OC1 Cells

**Figure 8:**
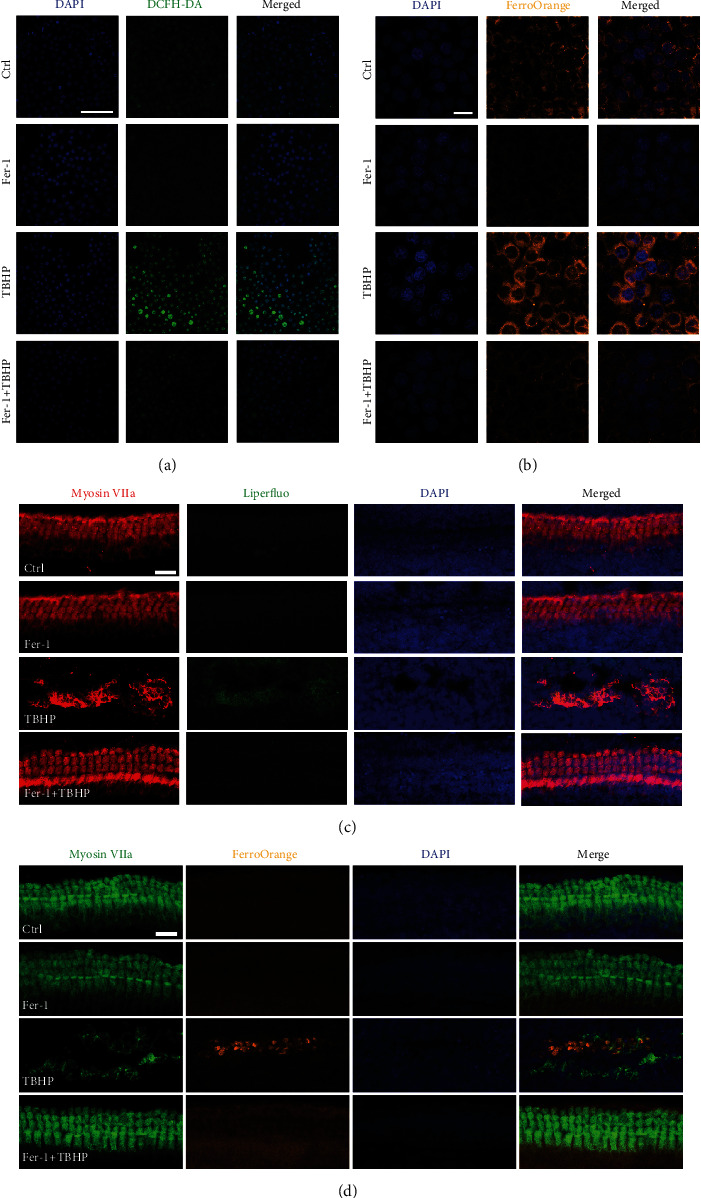
Treatment with Fer-1 alleviates TBHP-induced oxidative stress and iron accumulation in cochlear explants and HEI-OC1 cells. (a). Representative images of DCFH-DA staining in HEI-OC1 cells treated with TBHP and Fer-1. Scale bar = 100 *μ*m. (b). Representative images of FerroOrange staining in HEI-OC1 cells treated with TBHP and Fer-1. Scale bar = 20 *μ*m. (c). Representative images of Liperfluo staining in cochlear explants treated with TBHP and Fer-1. Scale bar = 20 *μ*m. (d). Representative images of FerroOrange staining in cochlear explants treated with TBHP and Fer-1. Scale bar = 20 *μ*m.

To verify the ability of Fer-1 against ROS generation and lipid peroxidation, DCFH-DA staining and Liperfluo staining were performed in HEI-OC1 cells and cochlear explants, respectively. As shown in Figure 8(a), the fluorescence intensity of DCFH-DA was significantly increased after TBHP treatment compared with controls. In contrast, the fluorescence intensity of DCFH-DA between the Fer − 1 + TBHP group and the control group was almost at the same level. In addition, the fluorescence intensity of DCFH-DA in the Fer-1 only group was markedly decreased, indicating its strong capacity of ROS scavenging. Excessive ROS leads to lipid peroxidation, which can be evaluated by Liperfluo staining. As shown in Figure 8(c), the fluorescence intensity of Liperfluo in cochlear explants was significantly decrease in the Fer − 1 + TBHP group compared with the TBHP groups, indicating that Fer-1 exerts protective effect by alleviating lipid peroxidation.

FerroOrange, a novel fluorescent probe that enables the labeling of intracellular Fe^2+^, was used to evaluate iron accumulation in HEI-OC1 cells and cochlear explants. As shown in Figure 8(b), the fluorescence intensity of FerroOrange was significantly increased after TBHP treatment compared with controls, indicating excessive iron accumulation induced by TBHP treatment. However, treatment with Fer-1 markedly reduced the FerroOrange fluorescence intensity in HEI-OC1 cells. Furthermore, we tested the iron concentration in cochlear explants treated with TBHP to induce oxidative stress damage. As shown in Figure 8(d), the intensity of FerroOrange fluorescence was significantly increased after TBHP treatment. The FerroOrange fluorescence intensity in the Fer-1 + TBHP group was markedly reduced compared with the TBHP only group. These findings suggested that oxidative stress damage may lead to ferroptosis in HEI-OC1 cells and cochlear explants. And inhibition of ferroptosis was able to ameliorate TBHP-induced damage to HEI-OC1 cells and cochlear explants.

### 3.8. Treatment with Fer-1 Mitigated Noise-Induced Cochlear Hair Cell Ferroptosis through TfR1 but Not GPX4 or ACSL4

**Figure 9:**
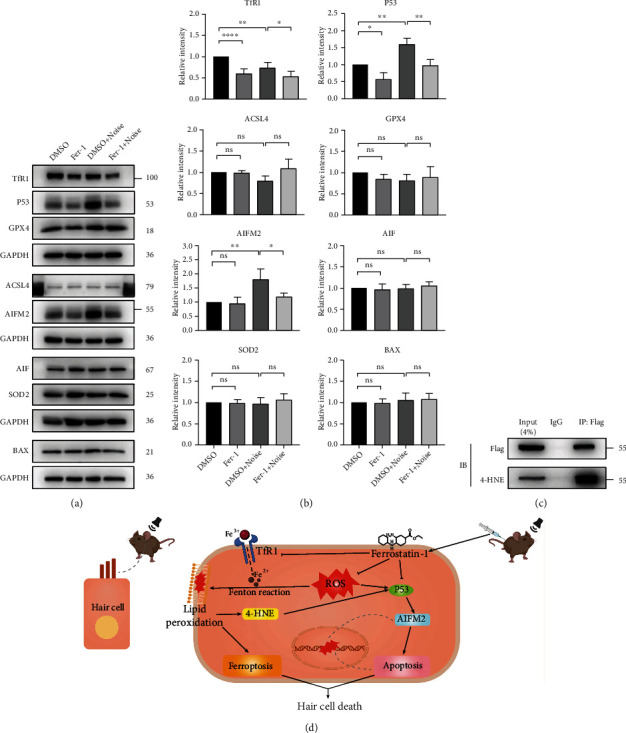
Fer-1 treatment exerts protective effects against NIHL partially by suppressing ferroptosis and apoptosis. (a) The expression levels of TfR1, P53, GPX4, ACSL4, AIFM2, AIF, SOD2, and BAX in the mouse cochlea exposed to different treatment combinations. (B) Semiquantification of the gray values of the TfR1, P53, GPX4, ACSL4, AIFM2, AIF, SOD2, and BAX bands. ^∗∗∗∗^*p* < 0.0001; ^∗∗^*p* < 0.01; ns: not significant. (c) HEK293 cells transfected with endogenously FLAG tagged P53 were lysed for immunoprecipitation with anti-FLAG and anti-4-HNE antibodies. (d) Ferrostatin-1 protects hearing by suppressing ferroptosis and apoptosis.

Ferroptosis is a recently discovered form of cell death characterized by lipid peroxidation and iron accumulation. The cochlea is a highly metabolic organ that produces abundant ROS when exposed to high-intensity noise. We confirmed that Fer-1 significantly reduced lipid peroxidation in the cochlea induced by noise. Therefore, we speculated that treatment with Fer-1 attenuates NIHL partially via inhibition of ferroptosis. As shown in Figures 9(a) and 9(b), we evaluated the expression of multiple key proteins involved in ferroptosis, such as GPX4, ACSL4, and TfR1. However, the levels of GPX4 and ACSL4 were not different among the four groups. As shown in Figure S4, we evaluated the ratio of GSH/GSSG and levels of glutathione in mouse cochleae. The results showed no statistical difference among the four groups. Moreover, we performed immunofluorescence staining for GPX4 and ACSL4 in frozen sections (Figure S4A and Figure. S5D). Quantification of relative GPX4 and ACSL4 immunolabeling intensity in grayscale showed no statistical difference among the four groups. In addition, the mRNA expression levels of GPX4 and ACSL4 were not different among the four groups (Figure S4B and Figure S5H). Notably, as shown in Figures 9(a) and 9(b), the level of TfR1 was significantly reduced after treatment with Fer-1 (DMSO vs. Fer-1, *p* < 0.0001). Treatment with Fer-1 also significantly reduced TfR1 levels after noise exposure (DMSO + noise vs. Fer − 1 + noise, *p* = 0.0204). However, the level of TfR1 was significantly reduced after noise exposure in the absence of Fer-1 (DMSO vs. DMSO + noise, *p* = 0.0025). As shown in Figure S5G, the mRNA level of TfR1 was significantly reduced after treatment with Fer-1 (DMSO vs. Fer-1, *p* = 0.0069) or noise exposure (DMSO vs. DMSO + noise, *p* = 0.0002), suggesting that Fer-1 may regulate the expression of TfR1 through transcriptional regulation. Furthermore, we tested the iron concentration in cochlear explants and HEI-OC1 cells treated with TBHP to induce oxidative stress damage. As shown in Figures.  8(b) and 8(d), the intensity of FerroOrange fluorescence was significantly increased after TBHP treatment. Pretreatment with Fer-1 markedly reduced the FerroOrange fluorescence intensity. These data indicated that Fer-1 alleviated NIHL partially via inhibition of TfR1-mediated ferroptosis (Figure 9(d)).

### 3.9. Treatment with Fer-1 Mitigated Noise-Induced Cochlear Hair Cell Apoptosis through the P53-AIFM2 Axis

AIFM2, also named apoptosis-inducing factor-like mitochondrion-associated inducer of death (AMID) or FSP1, is widely recognized for its ability to induce caspase-independent apoptosis [48]. Recently, AIFM2 was identified as an antiferroptotic protein because of its function as an oxidoreductase [37, 41, 42]. We first tested whether hair cells express this protein (Figure S2). Then, we evaluated whether the level of AIFM2 changes after noise exposure. As shown in Figures.  9(a) and 9(b), the level of AIFM2 was significantly increased after noise exposure (DMSO vs. DMSO + noise, *p* = 0.0057). Treatment with Fer-1 significantly reduced the level of AIFM2 after noise exposure (DMSO + noise vs. Fer − 1 + noise, *p* = 0.0265). We also performed immunofluorescence staining for AIFM2, and the results were similar to those of immunoblotting (Figure S5A). As shown in Figure. S5F, the mRNA level of AIFM2 was significantly reduced after treatment with Fer-1 (DMSO vs. Fer-1, *p* = 0.0375) or noise exposure (DMSO vs. DMSO+noise, *p* = 0.0187). These data indicated that AIFM2 plays a prodeath role rather than an antiferroptotic role during exposure to noise. Therefore, we evaluated the expression of its upstream target P53, a well-known proapoptotic protein. As shown in Figures.  9(a) and 9(b), the level of P53 was significantly reduced after treatment with Fer-1 (DMSO vs. Fer-1, *p* = 0.0400). After noise exposure, the level of P53 was significantly increased (DMSO vs. DMSO + noise, *p* = 0.0002), and treatment with Fer-1 significantly reduced the expression of P53 (DMSO + noise vs. Fer − 1 + noise, *p* = 0.0060). We also performed immunofluorescence staining for P53, and the results were similar to those of immunoblotting (Figure S5B). However, the mRNA level of P53 showed no statistical difference between the groups (Figure S5E). In addition, we evaluated the expression level of AIF, which shares significant homology with AIFM2. However, the level of AIF was not different among the four groups. SOD2 (Mn-SOD) is the only superoxide dismutase in mitochondria, playing an important role in scavenging ROS in mitochondria. However, the level of SOD2 was not different among the four groups. We also performed immunofluorescence staining for SOD2, and the results were similar to those of immunoblotting (Figure S5C). We also evaluated the expression level of BAX, which is crucial for mitochondrial apoptosis pathway. However, the level of BAX was not different among the four groups. In addition, the mRNA expression levels of SOD2, BAX, and AIF were not different among the four groups (Figure S5I, Figure S5J, and Figure S5K). 4-HNE is the major product of lipid peroxidation, so we explored whether 4-HNE overexpression influenced P53-AIFM2 pathway. As shown in Figure 9(c), we demonstrated that 4-HNE interacts with P53. These data indicated that noise induced cochlear hair cell apoptosis partially via the P53-AIFM2 pathway and that treatment with Fer-1 inhibited this process (Figure 9(d)).

## 4. Discussion

Fer-1 is a novel synthetic antioxidant with the ability to block lipid peroxidation and is therefore used as a ferroptosis inhibitor. In this study, the protective effect of Fer-1 against NIHL and the possible underlying mechanism were investigated in in vivo and in vitro models. We confirmed that treatment with Fer-1 alleviated noise-induced ABR threshold elevation and OHC loss in mice. However, we did not observe IHC loss, which was consistent with previous reports [11]. In addition, we found that Fer-1 administration reduced the loss of IHC ribbon synapses and the degeneration of ANFs caused by noise exposure. Furthermore, we verified that Fer-1 treatment protected hearing by scavenging ROS to alleviate oxidative stress, inhibiting ferroptosis by downregulating TfR1 expression, and diminishing apoptosis by regulating the P53-AIFM2 pathway (Figure 9(d)).

HCs are extremely important for hearing because of their mechanoelectrical transduction ability and ability to form auditory signals. However, HCs are vulnerable and nonrenewable in mammals. In the present study, noise exposure caused OHC loss in mice, while IHCs were intact after noise exposure. This may have been because the intrinsic antioxidant capacity of OHCs and IHCs is different [49]. In addition, we found that OHCs in the region corresponding to ~16 kHz were intact 14 d after noise exposure, while the ABR threshold was elevated by approximately 40 dB. This indicated that OHCs were unable to perform their normal functions despite not dying. The underlying mechanism by which Fer-1 alleviates the ABR threshold shift at 16 kHz may involve maintaining the normal function of OHCs instead of reducing OHC loss. Recent studies have shown that ribbon synapses, which are located between IHCs and SGNs, are the most vulnerable structures in the cochlea. Consistent with our and others previous reports [19, 46, 47], the number of paired ribbon synapses is markedly reduced by noise exposure. ANFs started degeneration once they disconnected from synapses, possibly because the peripheral neurites of SGNs lost the nutrition provided by synapses. We also observed that noise exposure caused ANF degeneration. Fer-1 treatment significantly reduced noise-induced ribbon synapse loss and ANF degeneration. This finding offers hope for the treatment of noise-induced hidden hearing loss (NIHHL), the main pathologic change of which is the loss of ribbon synapses [47].

Current theories related to noise-induced cochlear metabolic damage center on oxidative stress evoked by excessive noise stimulation. In our study, oxidative stress was evaluated by multiple methods, such as analysis of 4-HNE, 3-NT, DHE, MDA levels, Liperfluo staining, and DCFH-DA staining, and the results were consistent with previous studies [11, 14, 19, 20, 35]. An excessive amount of ROS in the cochlea, which is one of the most important features of ferroptosis, results from noise-induced lipid peroxidation through reactions with polyunsaturated fatty acids in the cell membrane. Previous studies have demonstrated that HEI-CO1 cells can undergo ferroptosis, and treatment with Fer-1 or Liproxstatin-1 (another inhibitor of ferroptosis) can reduce cell death induced by cisplatin or neomycin, respectively. In our study, we found that treatment with Fer-1 significantly reduced TBHP-induced HEI-OC1 cell death. In addition, some researchers have found that relieving ferroptosis may partially reverse neurodegeneration of the auditory cortex [33, 35, 50]. Therefore, we speculated that Fer-1 can attenuate NIHL via inhibition of ferroptosis. We first demonstrated the protective effect of Fer-1 on hearing. Then, we explored the underlying mechanism. GPX4, the most important glutathione peroxidase (GPX), has been proven to play a crucial role in protecting cells against ferroptosis caused by membrane lipid peroxidation [51]. We measured the mRNA and protein expression levels of GPX4 and glutathione content in the mouse cochlea after noise exposure in the presence or absence of Fer-1. However, there were no changes among the four groups. We then focused on ACSL4, the key enzyme in the regulation of lipid composition. ACSL4 has been proven to contribute to ferroptosis in ischemia/reperfusion [52]. The mRNA and protein expression levels of ACSL4 in the mouse cochlea were similar to those of GPX4. Then, we focused on iron, another key factor in ferroptosis. We found that the expression level of TfR1 in the mouse cochlea was markedly reduced by Fer-1 treatment, regardless of whether mice were exposed to noise. We observed that the mRNA level of TfR1 was decreased in the Fer-1 group compared with the DMSO control group, suggesting that Fer-1 may inhibit the transcription of TfR1 gene. Furthermore, the level of ROS after noise exposure was measured. We found that ROS were markedly eliminated by Fer-1 treatment. In addition, we studied oxidative stress in vitro in HEI-OC1 cells and cochlear explants. Our results demonstrated that Fer-1 markedly reduced hair cell loss, lipid peroxidation, and iron accumulation caused by TBHP. These data indicated that Fer-1 inhibited noise-induced hair cell ferroptosis by scavenging ROS and downregulating the expression of TfR1.

Excessive ROS also cause apoptosis. Mitochondria are considered the major ROS-producing organelles and are crucial proapoptotic mediators [53]. Our previous study demonstrated that cochlear mitochondrial dysfunction can be induced by noise exposure in a rat model [19]. Therefore, we speculated that Fer-1 can attenuate NIHL via inhibition of mitochondria-mediated apoptosis. In the present work, the expression level of AIFM2 was evaluated because AIFM2 is a caspase-independent apoptosis inducer. We found that the level of AIFM2 was robustly increased as a result of noise exposure and that this change was accompanied by auditory threshold elevation and OHC loss. However, AIFM2 mRNA did not increase after noise exposure (Figure S5F), suggesting that noise may inhibit the degradation of AIFM2 protein. Interestingly, AIFM2 was recently identified as an antiferroptotic protein because of its ability to reduce coenzyme Q10 levels to generate a lipophilic radical-trapping antioxidant [42]. What role does AIFM2 play in noise-induced hair cell loss? Some researchers have found that 4-HNE, a marker of lipid peroxidation, inactivate the NADH oxidoreductase function of AIFM2, facilitate its translocation from mitochondria and finally cause apoptosis in the heart tissues of mice [39]. In our study, noise exposure markedly increased the levels of 4-HNE and AIFM2 in the cochlear tissues of mice. Therefore, we speculated that 4-HNE may inhibit the degradation of AIFM2 and AIFM2 serves as an antiferroptotic mediator under normal conditions and becomes a proapoptotic mediator when an increase in 4-HNE levels in cochlear hair cells is induced by excessive noise-induced ROS. This hypothesis needs further investigation.

P53 is the upstream protein of AIFM2 and has multiple functions, including the induction of cell cycle arrest, senescence, and apoptosis. Recent studies have summarized the prooxidant and antioxidant activities of P53, illustrating the complexity of P53 in regulating cellular ROS metabolism [40, 54]. In our research, we found that the P53 protein level was markedly increased after noise exposure, but the mRNA level of p53 did not change (Figure S5E). Treatment with Fer-1 reduced the protein expression level of P53 in the presence or absence of noise. Moreover, we demonstrated that 4-HNE also interacts with P53. So, we speculated that 4-HNE adduction may affect ubiquitination degradation of P53, resulting in overexpression of P53 and apoptosis. This hypothesis needs further investigation. In addition to AIFM2, TfR1 is a downstream target of P53. Some researchers have revealed that TfR1 protein levels are decreased following induction of p53 expression [55]. Notably, we observed that the mRNA and protein levels of TfR1 were decreased in the noise exposure group compared with the DMSO control group. Considering the increase in the level of P53 caused by noise, the decrease in the level of TfR1 may be partially a result of the induction of P53 expression. In addition, P53 was found to suppress the transcription of SLC7A11 and enhance ALOX15 expression by activating SAT1, triggering ferroptosis consequently [54]. Taken together, our results and those of other researchers indicate that p53 plays dual roles in regulating hair cell death: it serves as an antiferroptotic factor via inhibition of TfR1 when ROS levels are moderate and becomes a proapoptotic and proferroptotic factor during ROS overload. Fer-1 promotes hair cell survival by scavenging ROS directly and inhibiting TfR1-mediated ferroptosis and P53-AIFM2 signaling pathway-mediated apoptosis.

There are several limitations to the present study. First, we only evaluated mouse hearing at 8, 16, and 32 kHz via ABR measurement. These three frequencies only cover approximately half of the cochlear epithelium (from 1 mm from the apex to 4 mm from the apex) and may not reflect the status of mouse hearing well. In future work, we will test the ABR at more frequencies and employ more methods to evaluate hearing, such as distortion product otoacoustic emissions (DPOAEs) and compound action potential (CAPs). Second, we used whole cochlear tissue homogenates to evaluate protein changes because we were unable to isolate hair cells due to technical difficulties. Finally, Fer-1 was administered systemically, and the concentration in the cochlea may have been low. In future studies, we will explore the feasibility of intratympanic (invasive) delivery to increase the concentration in the cochlea [56] . Furthermore, we want to explore the ability of novel hair cell-targeting nanoparticles or selectively deliver drugs within the inner ear.

## 5. Conclusions

In summary, our study is the first to explore the link between ferroptosis and apoptosis in NIHL. Noise-induced excessive ROS cause lipid peroxidation and activate the P53-AIFM2 signaling pathway in hair cells, leading to ferroptosis and apoptosis. Treatment with Fer-1 effectively protects hearing and alleviates the loss of OHCs, ribbon synapses, and ANFs induced by acoustic trauma. The mechanism of these protective effects may partially involve scavenging of ROS and inhibition of TfR1-mediated ferroptosis and P53-AIFM2 signaling pathway-mediated apoptosis. Our findings open new avenues for the treatment of NIHL.

## Figures and Tables

**Table 1:** Posthoc analysis of outer hair cell loss (Figure 2(c)).

Groups	Distance from apex (mm)	p-value	Symbol
DMSO + noise vs. Fer − 1 + noise	3.5	0.0006	^∗∗∗^
	4	<0.0001	^∗∗∗∗^
	4.5	<0.0001	^∗∗∗∗^
	5	<0.0001	^∗∗∗∗^

## Data Availability

The data presented in this study are available on reasonable request from the corresponding author.

## Abbreviations

3-NT: 3-Nitrotyrosine
4-HNE: 4-Hydroxynonenal
ABR: Auditory brainstem response
AIF: Apoptosis-inducing factor
AIFM2: Apoptosis-inducing factor mitochondria-associated 2
ANF: Auditory nerve fiber
BBN: Broadband noise
CCK-8: Cell counting kit
DMSO: Dimethyl sulfoxide
DHE: Dihydroethidium
EDTA: Ethylenediaminetetraacetic acid disodium salt
IHC: Inner hair cell
Fer-1: Ferrostatin-1
MDA: 3,4-Methylenedioxyamphetamine
NIHL: Noise-induced hearing loss
OHC: Outer hair cell
PBS: Phosphate buffered saline
PFA: Paraformaldehyde
PTS: Permanent threshold shifts
PL-PUFAs: Phospholipids with polyunsaturated acyl tails
ROS: Reactive oxygen species
SBE-*β*-CD: Sulfobutylether-*β*-cyclodextrin
SPL: Sound pressure level
TBHP: Tert-butyl hydroperoxide
TDT: Tucker Davis technology
TfR1: Transferrin receptor 1
RIPA: Radio-immunoprecipitation assay.

## Supplementary Materials

Supplementary Materials Figure S1: the baseline auditory threshold of the animals. The baseline auditory thresholds of the left ear of animals over 8-32 kHz evaluated via ABR testing. Data are presented as means ± SD. Figure S2: AIFM2 is expressed in cochlea. AIFM2 fluorescence is uniformly distributed in the cytoplasm. Scale bar = 100 *μ*m. HCs: hair cells; SV: stria vascularis; SGNs: spiral ganglion neurons. Figure S3: the chromatogram and mass spectrum of the perilymph sample. (A) Representative picture of the total ion current (TIC) chromatogram of the perilymph sample. (B) The tandem mass spectrum. The mass-to-charge ratio (*m*/*z*) of three daughter ions were 180.95, 152.90, and 134.95. (C) At 15 min, 1 h, and 3 h after injection, the concentration of the Fer-1 in perilymph of mouse cochlea was 2.077, 0.301, and 0.122 *μ*g/L, respectively. Figure S4: Fer-1 treatment or noise exposure have little effect on GPX4 expression and the content of GSH in cochlea. (A) Representative pictures of GPX4 (green) in frozen cochlear sections. Scale bar = 100 *μ*m. SV: stria vascularis; SGNs: spiral ganglion neurons; HCs: hair cells. (B) qRT -PCR verification of GPX4 mRNA expression levels in the cochlea of four groups of mice. (C) The GSH/GSSG ratio in the cochlea of four groups of mice. (D) The concentration of GSH (*μ*mol/L) in the cochlea of four groups of mice. (E) The concentration of GSSG (*μ*mol/L) in the cochlea of four groups of mice. (F) The concentration of glutathione (GSH and GSSG) (*μ*mol/L) in the cochlea of four groups of mice. Figure S5: Fer-1 treatment exerts protective effects against NIHL partially by suppressing ferroptosis and apoptosis. (A‑D) Representative pictures of AIFM2, P53, SOD2, and ACSL4 in frozen cochlear sections. Scale bar = 100 *μ*m. SV: stria vascularis; SGNs: spiral ganglion neurons; HCs: hair cells. (E‑K) qRT-PCR verification of P53, AIFM2, TfR1, ACSL4, SOD2, BAX, and AIF mRNA expression levels in the cochlea of four groups of mice. Table S1: primers for qRT-PCR analysis. The primer sequences for qRT-PCR analysis. The relative mRNA transcription levels were calculated by the 2^-*ΔΔ*CT^ method.
