# Supplementary material for: Treatment with the Ferroptosis Inhibitor Ferrostatin-1 Attenuates Noise-Induced Hearing Loss by Suppressing Ferroptosis and Apoptosis
Source: Oxid Med Cell Longev. 2022 Dec 7;2022:3373828. doi: 10.1155/2022/3373828 (PMC9750774; doi:10.1155/2022/3373828)
Supplement: Supplementary Materials — Figure S1: the baseline auditory threshold of the animals. The baseline auditory thresholds of the left ear of animals over 8-32 kHz evaluated via ABR testing. Data are presented as means ± SD. Figure S2: AIFM2 is expressed in cochlea. AIFM2 fluorescence is uniformly distributed in the cytoplasm. Scale bar = 100 μm. HCs: hair cells; SV: stria vascularis; SGNs: spiral ganglion neurons. Figure S3: the chromatogram and mass spectrum of the perilymph sample. (A) Representative picture of the total ion current (TIC) chromatogram of the perilymph sample. (B) The tandem mass spectrum. The mass-to-charge ratio (m/z) of three daughter ions were 180.95, 152.90, and 134.95. (C) At 15 min, 1 h, and 3 h after injection, the concentration of the Fer-1 in perilymph of mouse cochlea was 2.077, 0.301, and 0.122 μg/L, respectively. Figure S4: Fer-1 treatment or noise exposure have little effect on GPX4 expression and the content of GSH in cochlea. (A) Representative pictures of GPX4 (green) in frozen cochlear sections. Scale bar = 100 μm. SV: stria vascularis; SGNs: spiral ganglion neurons; HCs: hair cells. (B) qRT -PCR verification of GPX4 mRNA expression levels in the cochlea of four groups of mice. (C) The GSH/GSSG ratio in the cochlea of four groups of mice. (D) The concentration of GSH (μmol/L) in the cochlea of four groups of mice. (E) The concentration of GSSG (μmol/L) in the cochlea of four groups of mice. (F) The concentration of glutathione (GSH and GSSG) (μmol/L) in the cochlea of four groups of mice. Figure S5: Fer-1 treatment exerts protective effects against NIHL partially by suppressing ferroptosis and apoptosis. (A‑D) Representative pictures of AIFM2, P53, SOD2, and ACSL4 in frozen cochlear sections. Scale bar = 100 μm. SV: stria vascularis; SGNs: spiral ganglion neurons; HCs: hair cells. (E‑K) qRT-PCR verification of P53, AIFM2, TfR1, ACSL4, SOD2, BAX, and AIF mRNA expression levels in the cochlea of four groups of mice. Table S1: primers for qRT-PCR analysis. The pr [file 3373828.f1.docx]

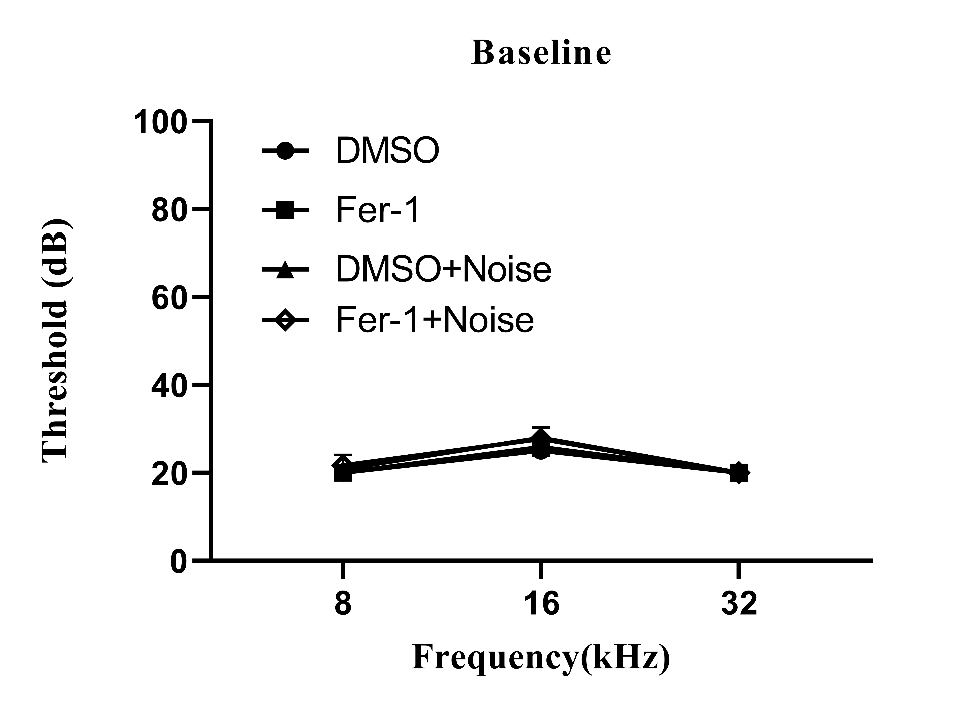


**Figure S1. The baseline auditory threshold of the animals.** The baseline auditory thresholds of the left ear of animals over 8-32 kHz evaluated via ABR testing. Data are presented as means ± SD.


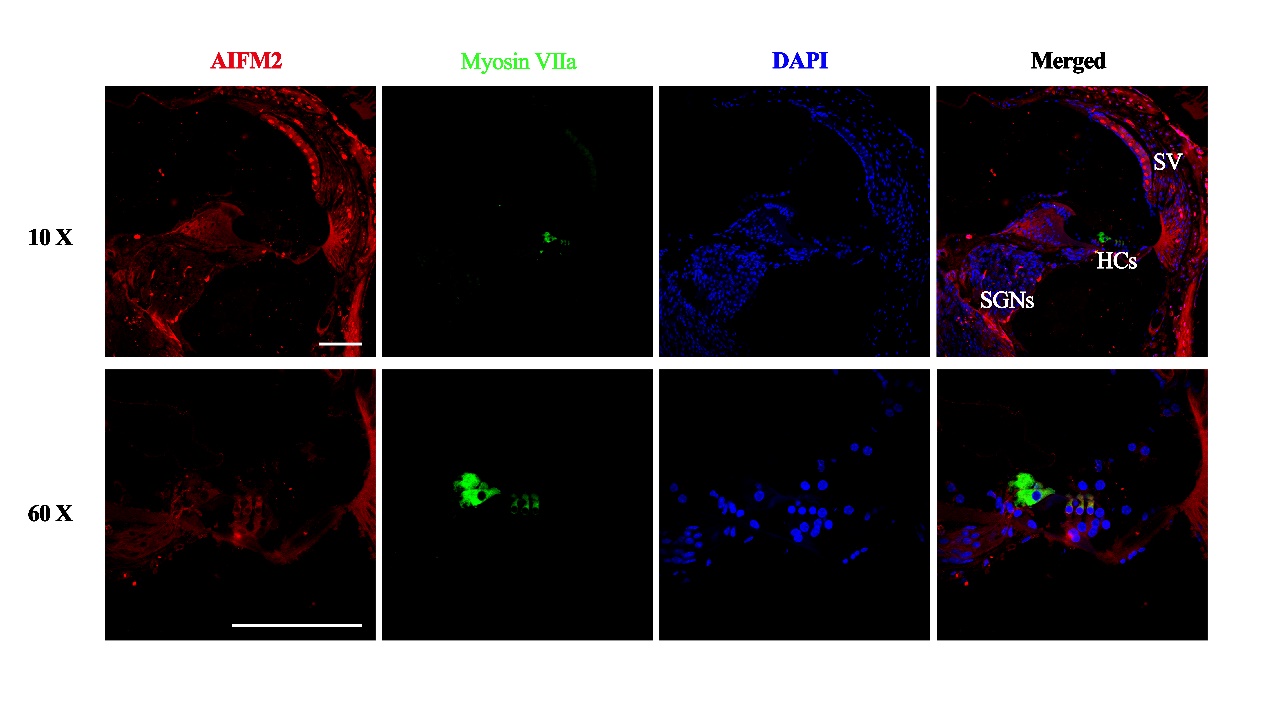


**Figure S2. AIFM2 is expressed in cochlea.** AIFM2 fluorescence is uniformly distributed in the cytoplasm. Scale bar = 100 µm. HCs: hair cells; SV: stria vascularis; SGNs: spiral ganglion neurons.


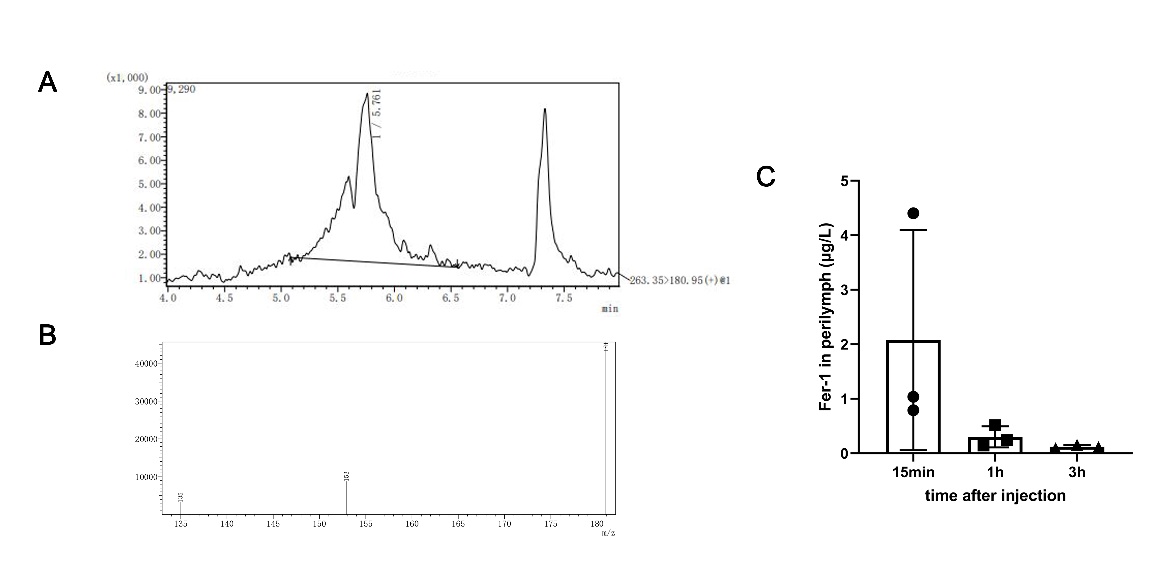


**Figure S3. The chromatogram and mass spectrum of the perilymph sample.** (A) Representative picture of the total ion current (TIC) chromatogram of the perilymph sample. (B) The tandem mass spectrum. The mass-to-charge ratio (m/z) of three daughter ions were 180.95, 152.90 and 134.95. (C) At 15min, 1h and 3h after injection, the concentration of the Fer-1 in perilymph of mouse cochlea was 2.077, 0.301 and 0.122 μg/L, respectively.


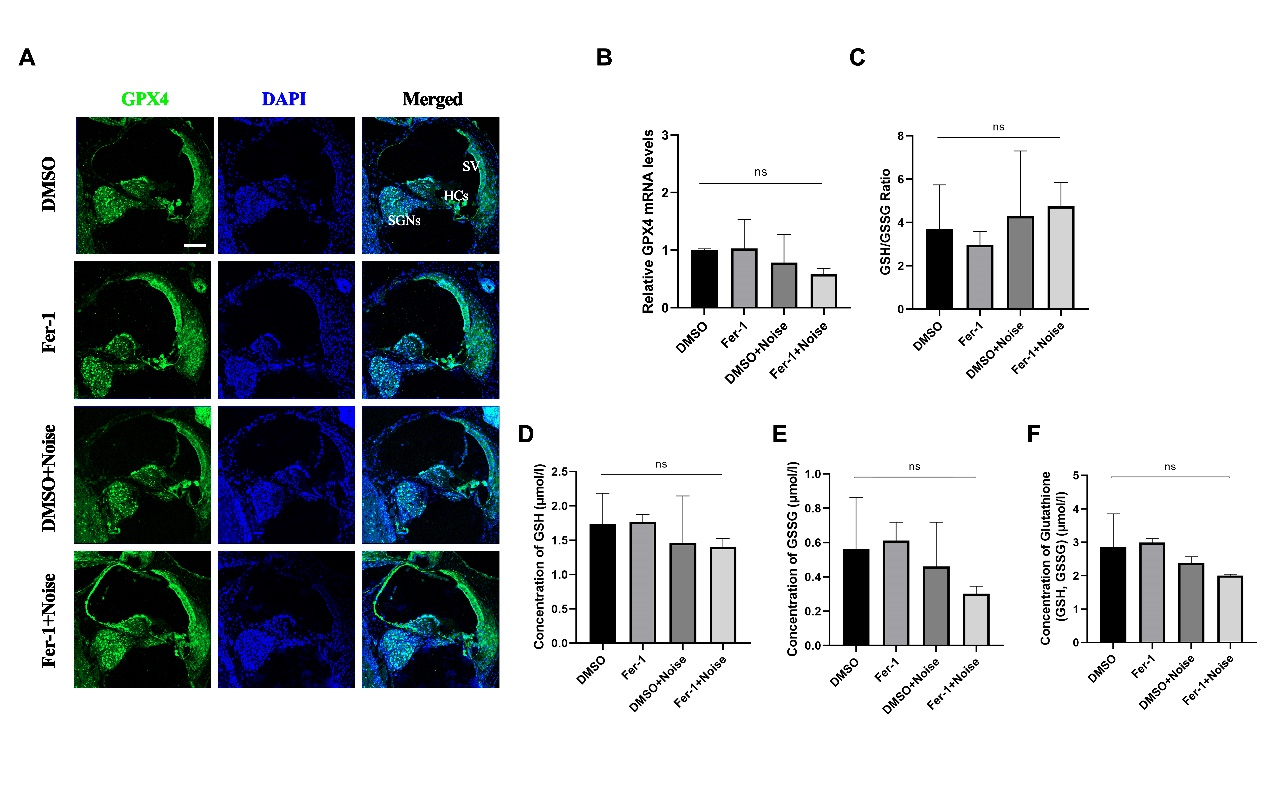


**Figure S4. Fer-1 treatment or noise exposure have little effect on GPX4 expression and the content of GSH in cochlea.** (A) Representative pictures of GPX4 (green) in frozen cochlear sections. Scale bar = 100 µm. SV, stria vascularis; SGNs, spiral ganglion neurons; hair cells, HCs. (B) qRT -PCR verification of GPX4 mRNA expression levels in the cochlea of four groups of mice. (C) The GSH/GSSG ratio in the cochlea of four groups of mice. (D) The concentration of GSH (μmol/L) in the cochlea of four groups of mice. (E) The concentration of GSSG (μmol/L) in the cochlea of four groups of mice. (F) The concentration of glutathione (GSH and GSSG) (μmol/L) in the cochlea of four groups of mice.


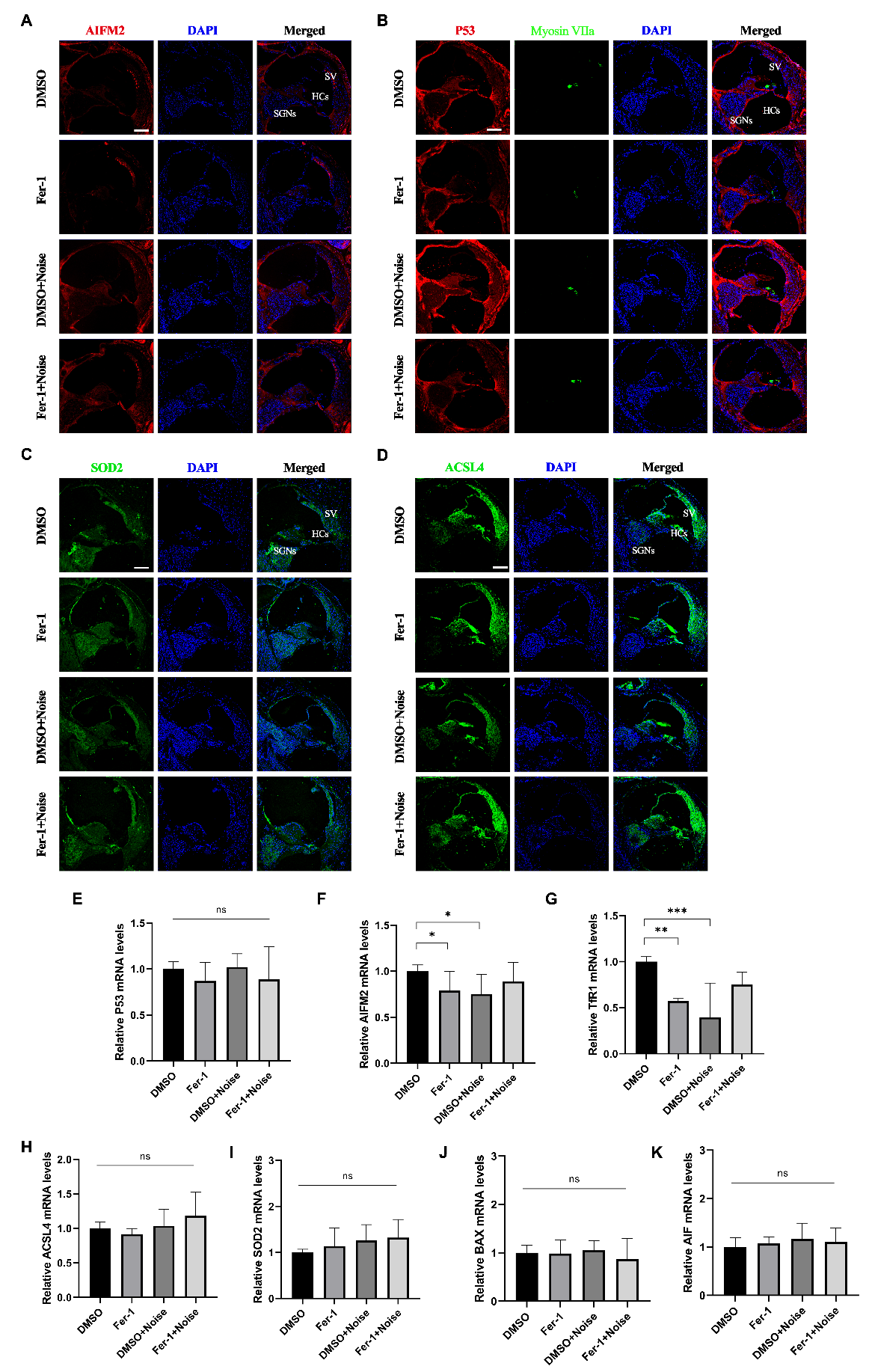


**Figure S5. Fer-1 treatment exerts protective effects against NIHL partially by suppressing ferroptosis and apoptosis.** (A-D) Representative pictures of AIFM2, P53, SOD2 and ACSL4 in frozen cochlear sections. Scale bar = 100 µm. SV, stria vascularis; SGNs, spiral ganglion neurons; hair cells, HCs. (E-K) qRT -PCR verification of P53, AIFM2, TfR1, ACSL4,SOD2,BAX and AIF mRNA expression levels in the cochlea of four groups of mice.

**Table S1. Primers for qRT-PCR analysis**

| Name | Sequence (5’-3’) |
| --- | --- |
| TfR1 | F: TGGAGACTACTTCCGTGCTAC |
|  | R: TCCACTAAAGCTGAGAGGGTG |
| GPX4 | F: GTTTCGTGTGCATCGTCACC |
|  | R: GGGCATCGTCCCCATTTACA |
| P53 | F: TCCGAAGACTGGATGACTGC |
|  | R: GATCGTCCATGCAGTGAGGT |
| AIFM2 | F: ACCGCAGTGCATTTGAGAGTA |
|  | R: GGTATCGGCACAGTCACCAA |
| ACSL4 | F: CCAGTGGCAGACTCGTAGC |
|  | R: AGCCAGCAATAAAGTACACAGAT |
| AIF | F: TCCAGAGGCCGAAACAGAG |
|  | R: CCGTGGTATTCGACCCGTTC |
| SOD2 | F: CTGGAACCAGTTGCCTGGAA |
|  | R: CTCCAGCAACTCTCCTTTGGG |
| BAX | F: AAACTGGTGCTCAAGGCCC |
|  | R: AGCCACCCTGGTCTTGGAT |
| ACTIN | F: GTCCCTCACCCTCCCAAAAG |
|  | R: GCTGCCTCAACACCTCAACCC |
